# Bioinspired Microstructured Polymer Surfaces with Antireflective Properties

**DOI:** 10.3390/nano11092298

**Published:** 2021-09-04

**Authors:** Alexandre Emmanuel Wetzel, Nuria del Castillo Iniesta, Einstom Engay, Nikolaj Kofoed Mandsberg, Celine Schou Dinesen, Bilal Rashid Hanif, Kirstine Berg-Sørensen, Ada-Ioana Bunea, Rafael Taboryski

**Affiliations:** 1 National Centre for Nano Fabrication and Characterization (DTU Nanolab), Technical University of Denmark, Ørsted Plads 347, 2800 Kongens Lyngby, Denmark; awet@dtu.dk (A.E.W.); nurin@dtu.dk (N.d.C.I.); einen@dtu.dk (E.E.); s173957@student.dtu.dk (C.S.D.); s173968@student.dtu.dk (B.R.H.); rata@dtu.dk (R.T.); 2 Center for Intelligent Drug Delivery and Sensing Using Microcontainers and Nanomechanics (IDUN), Technical University of Denmark, Ørsted Plads 345C, 2800 Kongens Lyngby, Denmark; nikoma@dtu.dk; 3 Department of Health Technology (DTU Health Tech), Technical University of Denmark, Ørsted Plads 345C, 2800 Kongens Lyngby, Denmark; kibs@dtu.dk

**Keywords:** 3D printing, antireflective, biomimetic, polymer microstructures, super black, two-photon polymerization

## Abstract

Over the years, different approaches to obtaining antireflective surfaces have been explored, such as using index-matching, interference, or micro- and nanostructures. Structural super black colors are ubiquitous in nature, and biomimicry thus constitutes an interesting way to develop antireflective surfaces. Moth-eye nanostructures, for example, are well known and have been successfully replicated using micro- and nanofabrication. However, other animal species, such as birds of paradise and peacock spiders, have evolved to display larger structures with antireflective features. In peacock spiders, the antireflective properties of their super black patches arise from relatively simple microstructures with lens-like shapes organized in tightly packed hexagonal arrays, which makes them a good candidate for cheap mass replication techniques. In this paper, we present the fabrication and characterization of antireflective microarrays inspired by the peacock spider’s super black structures encountered in nature. Firstly, different microarrays 3D models are generated from a surface equation. Secondly, the arrays are fabricated in a polyacrylate resin by super-resolution 3D printing using two-photon polymerization. Thirdly, the resulting structures are inspected using a scanning electron microscope. Finally, the reflectance and transmittance of the printed structures are characterized at normal incidence with a dedicated optical setup. The bioinspired microlens arrays display excellent antireflective properties, with a measured reflectance as low as 0.042 ± 0.004% for normal incidence, a wavelength of 550 nm, and a collection angle of 14.5°. These values were obtained using a tightly-packed array of slightly pyramidal lenses with a radius of 5 µm and a height of 10 µm.

## 1. Introduction

Antireflective (AR) coatings are typically applied to the surface of various optical elements in order to reduce reflection and therefore improve efficiency. AR coatings have been around for more than a century, with the simplest form dating back to Lord Rayleigh’s 1886 tarnished glass [1]. Different approaches for obtaining AR coatings exploit index-matching, interference, or absorbing phenomena.

When the directional reflectance of a surface is below 0.5% and all light is absorbed by the bulk material, that surface can be considered super black [2]. Super black surface treatment was first developed by Brown et al. at the National Physical Laboratory in the UK in 2002 [3] and soon gained significant interest, both in academia and in industry. The super black surface was prepared by the chemical etching of an electroless-deposited nickel-phosphorus alloy, which reflected <0.4% of light in the visible region [3]. Since then, scientists have been competing in a race to produce the blackest material, with some of the most successful examples being black silicon [4,5,6], Vantablack, i.e., Vertically-Aligned carbon NanoTube Arrays [7], dark chameleon dimers based on gold nanoparticles [8], and a more recent carbon nanotube-based material developed at MIT [9]. Although extremely valuable, these solutions usually require complicated fabrication procedures and post-application treatments, as they are based on nanoparticles and/or nanostructures.

Structural colors also appear in nature, so another interesting approach for developing AR coatings is biomimicry. In nature, several organisms have been studied for their structural AR properties. There are many reports of combinations of micro- and nanostructures (hierarchical structures) that produce low to very low reflectance, for example velvet black snake scales [10], glasswing butterflies [11], viola petals [12], or rose petals [13]. Viola petals and rose petals have already been successfully replicated using a polydimethylsiloxane (PDMS) mold and ultraviolet (UV) curing resist, leading to a 1% reflectance close to normal incidence and 5% reflectance at an angle of incidence of 80°, respectively. Probably the most well-known example of nanostructure-only based AR materials are moth-eye AR structures, which are formed by corneal nipples arranged in a highly ordered hexagonal array, where the spacing between individual structures ranges from 180 to 240 nm [14]. The presence of the sub-wavelength nanostructures enables a gradual change of the effective refractive index between the air and the moth-eye medium, leading to excellent AR properties known as the “moth-eye effect” [9,10]. Bioinspired moth-eye patterns have been successfully fabricated using micro- and nanofabrication methods and employed as AR coatings [15,16,17,18,19,20,21]. Replication of such nanostructures was carried out on several materials, including silicon [19,20], vanadium dioxide [21], chalcogenide glass [15], and transparent polymer foils [20], where measurements showed a reflectance of less than 0.5% for wavelengths between 500 to 900 nm, an absorption of 99% in the visible range, a transmittance above 70% for wavelengths between 3.3 to 13 µm, and above 90% in the visible range, respectively. Both hierarchical and nanostructures with AR properties rely mainly on the interaction of light with subwavelength structures, enabling a generally low reflectance over a large wavelength range. Interestingly, recent studies from Harvard highlight two other types of AR structures that lead to super black coloring in nature: barbule microstructures on birds of paradise [22] and cuticular bumps on peacock spiders [2]. The two recent publications provide detailed information on the shape of such natural super black microstructures and the mechanisms behind the observed super black effects. Unlike the hierarchical and nanostructures with AR properties discussed above, the AR structures on birds of paradise and peacock spiders are rather large, with their smallest features being micrometer-sized. The peacock spider AR structures have lens-like shapes, and their main advantages over subwavelength structures reside in their ability to considerably increase the transmitted average path length in active underlying layers, such as the absorbing or photovoltaic layers, as well as their relatively easier fabrication [23]. Although it should prove extremely valuable, replication of such structures has not yet been demonstrated. This work therefore represents the first experimental replication of AR structures inspired by the peacock spider’s natural cuticular bumps.

The super black naturally occurring patches in peacock spiders consist of microarrays of tightly-packed microlenses, with individual lenses having a diameter of approximately 4–13 µm, on top of an absorbing melanin layer [2]. Each microlens has a superellipsoidal shaped surface, described by Equation (1), where $R_{0}$ is the characteristic structure size, $R_{0}h_{0}$ is the height, $e_{0}$ is the elongation, and N∈[1; 2] is the shape factor, with N=1 describing a near-pyramidal shape and N=2 describing an ellipsoid [2]:

(1) $$z\left(x,y\right)=R_{0}h_{0}{\left[1-{\left|\frac{x}{R_{0}}\right|}^{N}-{\left|\frac{y}{R_{0}e_{0}}\right|}^{2}\right]}^{\sqrt[{-2}]{2N}}$$

All the shape parameters from Equation (1) can be adjusted in order to identify the most promising superellipsoidal microarrays for AR coatings. Rapid prototyping is the best approach for small-scale fabrication when parameter optimization is essential. Given the fact that the peacock spider’s cuticular bumps are only several micrometers in size, a rapid prototyping technique with sub-micrometer resolution is required. Direct laser writing (DLW) using 2PP was pioneered in the late 1990s [24] and is the only 3D printing technique that can directly write sub-micrometer features. Early commercial 2PP equipment was quite slow, ~0.1–30 mm/s, as it required extremely precise physical displacement of the 3D printing stage using stepper motors or a piezoelectric stage [25]. Recent technological advancements have supplemented stage displacement with beam displacement, using digital miromirror devices or galvanometers, enabling printing speeds up to 400 mm/s [25], thus making the technology much more suitable for rapid prototyping parameter sweeps. Currently, 2PP enables full 3D design freedom with a printing resolution of 100–200 nm in the XY plane, and a 100 µm^2^ pattern can be printed in a few minutes [26,27]. Therefore, we selected 2PP 3D printing as fabrication technique for our study.

In this work, we demonstrate the fabrication of AR biomimetic micropatterns inspired from the peacock spider’s natural cuticular bumps. Using 2PP 3D printing, we fabricated microarrays of microlenses designed according to Equation (1) and measured their specular reflectance and transmittance. Several structure geometries were investigated by changing the shape parameters in Equation (1) in order to optimize the structure’s AR properties. In addition, flat samples were printed to confirm that the reduction in specular reflectance is due to the structure’s geometries.

## 2. Materials and Methods

### 2.1. Fabrication

#### 2.1.1. File Generation for 3D Printing

In order to print the wanted structures from Equation (1), the printer software is first provided with a code that it can read. For this purpose, the first step is to generate an .STL file containing the 3D shape information. As most computer-aided design (CAD) software cannot handle generating a structure based on Equation (1), the meshes and .STL files were generated directly in MATLAB^®^. For this, a single microlens was first created and then replicated and displaced to form a tightly packed array. For $e_{0}=1$ and N=2, the structures formed are hemispheres and the lattice is hexagonal. For other sets of parameters, the lattice was modified to obtain maximum surface coverage, while avoiding overlapping structures. Additional details of the generation process, as well as examples of obtained single structures, are found in Section S1 of the Supporting Information (SI).

The .STL files generated using MATLAB^®^ were subsequently imported into the DeScribe software (Nanoscribe, Karlsruhe, Germany) where the code for printing, i.e., the job file, can be generated. Several parameters can be controlled in DeScribe, such as the slicing and hatching distances, the printing trajectory, the number of contour lines, or the laser power and scan speed. This software then converts the .STL file into a .GWL job file for the 3D printer. Additional information on the printing parameters is given in Section S2 of the SI.

#### 2.1.2. Two-Photon Polymerization Printing

The structures were printed using the Nanoscribe Photonic Professional GT+ system (Nanoscribe, Karlsruhe, Germany). This system makes use of the 2PP fabrication technique to reach a lateral resolution < 200 nm and an axial resolution < 700 nm [28]. The system employs a 780 nm Ti-Sapphire laser with a 150 fs pulse durations at 80 MHz repetition rate [26], and can print either by scanning the laser over the field of view (FOV) of the objective lens with galvo mirrors or by moving the sample using a piezoelectric stage. The structures were printed on a 30 mm Ø borosilicate glass coverslips using the IP-L 780 polyacrylate photoresist (Nanoscribe, Karlsruhe, Germany) and a Plan-APOCHROMAT 63×/1.40 Oil DIC objective (Carl Zeiss, Oberkochen, Germany). In order to avoid stitching errors, the microstructures were arranged in microarrays corresponding to a square with a side length below 140 µm, or a hexagon with a side length below 100 µm. The sizes were selected so that the microarrays could be inscribed in the maximum effective writing field for this microscope objective, which is a circle with a diameter of 200 µm. Several microarrays were printed on the same substrate. Individual micro arrays were printed using the galvo mirrors to scan the laser beam, after which the stage was displaced to a new area on the substrate using the piezoelectric motors.

### 2.2. Characterization

#### 2.2.1. SEM Imaging

The printed structures were coated with a layer (~15 nm) of gold in a sputter coater (Cressington Scientific Instruments, Watford, UK) and then imaged on a Zeiss Supra 40 VP (Carl Zeiss, Oberkochen, Germany) scanning electron microscope (SEM). All images were acquired in high vacuum mode using an acceleration voltage of 1.0 to 1.5 kV and the secondary electron detector.

#### 2.2.2. Optical Reflectance and Transmittance Measurements

**Figure 1:**
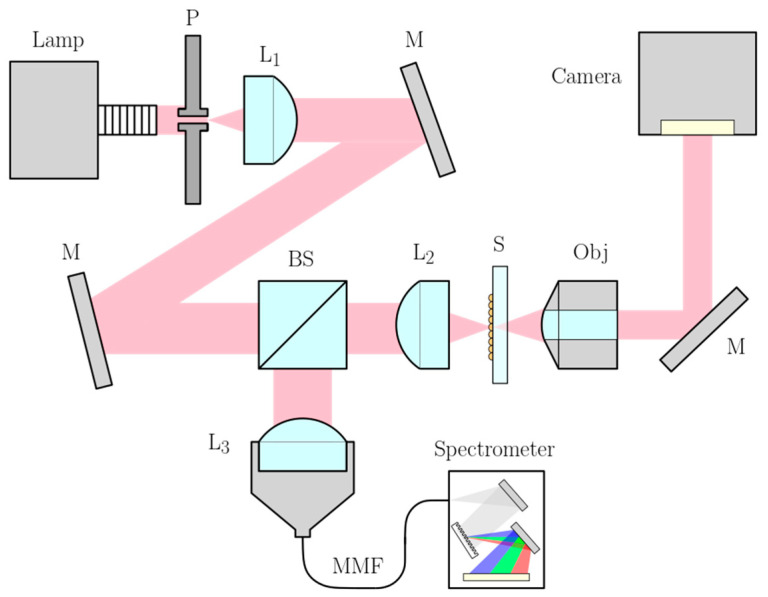
Optical reflectance and transmittance measurement setup: P: pinhole, L: lens, M: mirror, BS: beamsplitter, S: sample, Obj: microscope objective, MMF: multimode fiber.

The 3D micropatterns’ reflectance and transmittance were measured using the optical setup shown in Figure 1. A broadband halogen lamp (Euromex LE.5210, Arnhem, The Netherlands) was used for the light source, and a pinhole (Thorlabs P100D, ∅=100 µm, Newton, MA, USA) was imaged onto the samples using lenses L1 and L2 (f = 100 mm). A 30:70 beamsplitter (Thorlabs BS019) split the beam to achieve both spectral information as well as imaging modality. For the former, the lens, L3, (Thorlabs F810SMA-543, f = 34.74 mm) collected the reflected light from the sample and focused it into a multimode fiber (MMF), which guided the collected reflected light into a spectrometer (Ocean Insight USB2000+VIS-NIR-ES, Orlando, FL, USA). For imaging, the transmitted light was collected by a microscope objective (Olympus PLN20X) and imaged directly onto a camera (Basler acA1920-40uc). Our measurements were made at normal incidence and with total collection angles of 14.5° and 47.2° for the reflectance and transmittance, respectively.

The reflected light was characterized for each sample on the spectrometer and normalized by a reference spectrum to obtain their characteristic reflectance. In order to acquire the reference spectrum, the samples were replaced by a broadband mirror (Thorlabs BB1-E02). In addition, a spectrum was acquired with the light source off to take into account potential noise sources. The reflectance was then calculated as:

(2) $$R=\frac{I_{s}-I_{dark}}{I_{ref}-I_{dark}}$$

where $I_{s}$, $I_{ref}$, and $I_{dark}$ are the measured sample intensity, reference intensity, and intensity without the light source, respectively. In a similar way, the average spectral transmittance was characterized by imaging the samples onto a camera and measuring the average intensity ratio between the sample image and reference image over a region of interest (ROI) defined by the beam. This time, the reference image was acquired after removing the samples from the setup, and an image was taken with the light source off to remove potential noise. The transmittance was then calculated as:

(3) $$T=\frac{\bar{I_{s,ROI}}-\bar{I_{dark,ROI}}}{\bar{I_{ref,ROI}}-\bar{I_{dark,ROI}}}$$

where $\bar{I_{s,ROI}}$, $\bar{I_{ref,ROI}}$, and $\bar{I_{dark,ROI}}$ correspond to the sample, reference, and dark average intensity over the ROI, respectively. More information on the procedure to obtain the reflectance and average transmittance from the raw signals can be found in Section S3 of the SI.

## 3. Results

### 3.1. 3D Printed Microarrays

**Figure 2:**
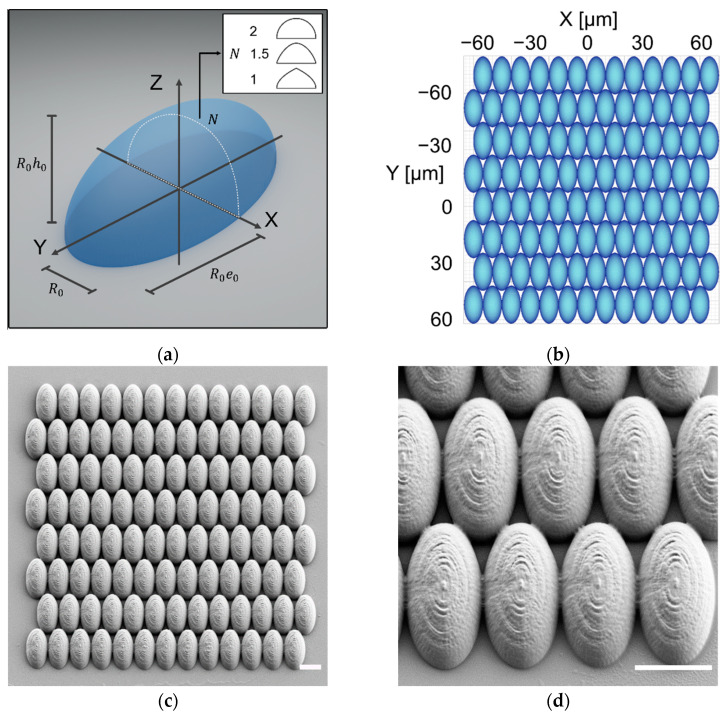
Microstructure fabrication process, from theoretical surface equation to 2PP 3D printing in the polyacrylate resin IP-L 780. (**a**) Single structure 3D representation with parameters from Equation (1) ($R_{0}=5\;µm$, $h_{0}=1$, $e_{0}=2$, and N=2); (**b**) 2D view of the microstructure array after importing the .STL file in the Describe software and preparation for 3D printing; (**c**) Scanning electron micrograph of the full printed array; (**d**) Scanning electron micrograph enlarged view of the singular elements of the array. The scale bar in (**c**,**d**) is 10 µm.

Each microlens array was printed in a polyacrylate resin according to a different set of structural parameters: $R_{0}$, $h_{0}$, $e_{0}$, and N. Before investigating all the parameters, hemispheres were first printed with varying characteristic structure sizes, $R_{0}$, ranging from 2 to 10 µm. Hemispheres can be obtained directly from Equation (1) by setting $h_{0}=1$, $e_{0}=1,$ and N=2. In a second step, $R_{0}$ was set to 5 µm and the other parameters were individually varied from 1 to 2, while keeping the remaining parameters the same as for the hemisphere. Finally, two more structures were printed based on the parameters of the least reflective samples obtained, as previously described. In addition to this, flat structures with different thicknesses from 2 to 10 µm were also printed in order to assess the impact of the 2-photon-induced surface roughness on the reflectance and transmittance. An example of the full process, from .STL file design to obtaining the printed structures, is shown in Figure 2. The SEM pictures in Figure 2c,d show that the printed arrays were tightly packed. The structures shape fitted well with the theoretical one, apart from small discretization artifacts stemming from both the Matlab .STL file and the ~200 nm resolution of the printer. The full list of printed structures according to the parameters tested can be found in Section S4 of the SI.

### 3.2. Reflectance and Transmittance Measurements

**Figure 3:**
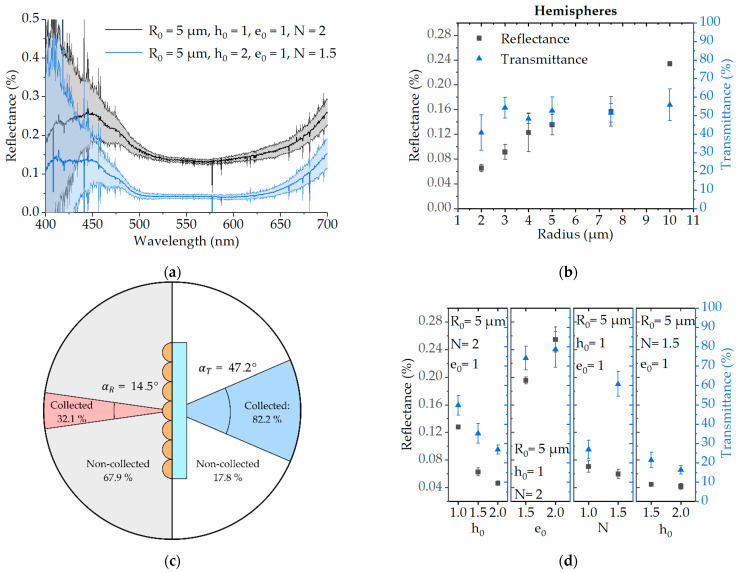
Structure’s reflectance and transmittance characteristics as a function of the design parameter from Equation (1). (**a**) Reflectance spectra of the most and least reflective structures with error and corresponding shape parameters; (**b**,**d**) average reflectance (at λ=550 nm) and transmittance of structures printed with corresponding error: (**b**) Hemispheres with varying radius $R_{0}$ ($h_{0}=1$, $e_{0}=1$ and N=2), (**d**) structures with varying height factor, $h_{0}$, elongation factor, $e_{0}$, pyramidal factor, N, and combinations of these parameters; (**c**) Collection angles and percentage of collected and transmitted light intensity estimation based on calculation in Section S5 (for a hemisphere with $R_{0}=5$ µm and at λ=550 nm). All errors in (**a**,**b**,**d**) correspond to 1σ.

The measured reflectance response from the different samples is found to be rather flat, from 500 nm to 650 nm, with no particular features, while it increases below 500 nm and above 650 nm. While the increase in reflectance below 500 nm can be expected, both from the collection of more diffracted light and the refractive index change at lower wavelengths [29], we assume that the intensity increase at wavelengths greater than 650 nm could be due to measurements error caused by a low signal from the light source. Therefore, in order to make it easier to compare different samples, we choose 550 nm as a representative wavelength in the flat part of the spectrum. An example of the measured spectra can be seen in Figure 3a.

An initial screening showed that the printed hemisphere’s transmittance is very similar for different radius sizes, while the reflectance decreases with the radius (see Figure 3b). Because the acceptance angle for the transmittance measurements is much larger than the acceptance angle for the reflectance measurements, this suggests that the light interacting with the structures is mainly subject to diffraction. Addionally, as opposed to strongly absorbing super black materials, our photoresist here is rather transparent. Therefore, ideal properties for an AR coating with such a microstructured material would be to minimize reflectance in the normal direction, while still providing high transmittance. Although all of our measured reflectances were below 0.5% and therefore the corresponding structures have promising AR properties, printing the smallest structures here would cause two kinds of issues. Firstly, the smallest structures promote diffraction as compared to the larger ones. Secondly, printing smaller structures induces larger discrepancies compared to the smooth theoretical structures, due to the aforementioned discretization and fabrication artifacts. Finally, the larger structures are more suitable for further large-scale replication. For all these reasons, we opted to use a characteristic structure size of $R_{0}=5$ µm before screening with the other parameters.

To quantify the effect of diffraction in our setup when using structures with $R_{0}=5$ µm, we simulated the diffraction efficiency of such hemispheres using the procedure detailed in Section S5 of the SI. Even though our collection angles are relatively small for both the reflectance and transmittance, we show that we collect approximately 17.8% and 50.4% of the total reflected and transmitted light, respectively (see Figure 3c). This supports our strategy for printing structures that are large enough so that they do not diffract too much of the incoming light.

In the second screening, we varied the values of $h_{0},$ $e_{0}$, and N from 1 to 2, while keeping the other parameters the same as for hemispheres ($h_{0}=1$, $e_{0}=1$, and N=2). This can be observed in Figure 3d. Overall, we see that increasing $h_{0}$ or using values of N<2 leads to a smaller reflectance and smaller transmittance, while increasing $e_{0}$ leads to larger reflectance and transmittance. Increasing $h_{0}$ is effectively making the structures aspect ratio larger, while decreasing N is making the structures more pyramidal. The reduction of reflectance and transmittance in this case could therefore be attributed to the larger angles of incidence on the structure’s surface. Similarly, the increase in reflectance with increasing values of $e_{0}$ can be attributed to the “flattening” of the structures, as well as to greater space between individual structures in the fabricated microarray. For N=1.5, the transmittance remains quite large, while the reflectance decreases, which is why we consider this shape factor of 1.5 optimal. Finally, we printed two additional samples, one with $h_{0}=1.5$ and one with $h_{0}=2$, where N=1.5 and $e_{0}=1$. This lead to the lowest reflectance measured overall, with a reflectance of 0.045 ± 0.002% and 0.042 ± 0.004%, respectively. However, these structures also had the lowest transmittance from all the printed samples: 21.4 ± 4.0% and 16.3 ± 2.2%, respectively.

### 3.3. Effect of the Printed Geometries

**Figure 4:**
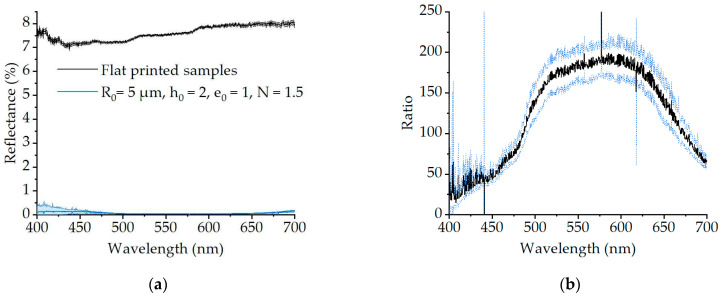
Effect of the printed geometries on the reflectance spectra. (**a**) Average reflectance spectra of the flat printed samples and of the optimized microlens array sample, with respective error of 1σ; (**b**) Ratio between the flat and optimized structure’s reflectance with blue lines showing error of 1σ.

The measured absolute reflectance and transmittance in this study are influenced both by the printed material and by the printed structure’s geometry. In order to isolate the effect of the structure’s geometry from that of the material, we fabricated a set of uniform flat structures to serve as a control. The height of the uniform flat structures ranged from 2 to 10 µm, corresponding to the hemispheres’ $R_{0}$ parameter shown in Table S2 of the SI. The reflectance of the control structures was characterized in the same way as for all other samples, and it can be considered constant for all tested heights (data not shown). Therefore, we only considered the average reflectance spectrum from the different samples in the following results. As shown in Figure 4a, the reflectance for the flat 3D printed structures is relatively featureless. In comparison, the reflectance for the microlens array sample is significantly lower. The ratio between the two spectra then shows that we obtain a decrease in reflectance of 150 to 200 times in the middle of the visible spectrum (see Figure 4b). This confirms that the antireflective effect observed in our experiments is due to the presence of the microstructures, rather than being caused by the employed material or by the surface roughness inherent to the 2PP 3D printing fabrication technique.

## 4. Discussion

As mentioned in the introduction, recent studies have reported AR super black microscale-only structures in birds of paradise and peacock spider [2,22]. While the feather structures from birds of paradise are quite complex, the peacock spider’s structures are made from packed arrays of superellipsoidal microlenses, as modelled in Equation (1), sitting on top of an absorbing layer. Our results show a good correspondence with the reflectance, both measured and simulated by McCoy et al. [2]. For example, our measurements confirm the predicted drop in reflectance for increasing $h_{0}$ values, for values of N<2, and an increase in reflectance for increasing $e_{0}$ values. In addition to this, we estimate that we collect 17.8% of the total reflected light when characterizing hemispheres with $R_{0}=5$ µm in our measurement setup. This would correspond to having a total or diffuse reflectance of 0.79% for this radius, at a wavelength of 550 nm. In their simulations, McCoy et al. obtained around 0.45% reflectance for the total reflected light with the same shape parameters [2]. We obtained in our measurements a reflectance value that is approximately double that of the value predicted by McCoy et al. [2]. This fits very well with the fact that, in their simulations, only one interface undergoes reflection, while two interfaces contribute to the signal in our experiments. Furthermore; our optimized structures showing minimum reflectance were obtained using parameters very similar to those encountered in nature for the Maratus Karrie peacock spider species. More specifically, the optimal structures described herein were fabricated using $h_{0}=2$ and $e_{0}=1$, with $R_{0}=5$ µm, whereas the cuticular bumps on Maratus Karrie species were measured to have $h_{0}\approx 2.1$, $e_{0}\approx 1.2$, and $R_{0}\approx 2.9$ µm.

Because the 2PP printed structures include small artifacts due to the printer’s resolution, further improvement could be implemented by, e.g., the use of thermal reflow to smoothen the surface of the fabricated microstructures [30,31].

Compared to nanoscale structures that can achieve relatively low diffuse or total reflectance, microscale structures will inevitably diffract the light. However, our study confirms here that the printed structures can still achieve very low specular reflectance for normally incident light. In addition, the specular transmittance and reflectance is tunable with microlens geometry. For example, we show that transmittance can be increased with increasing $e_{0}$ values, and the reflectance can be decreased with increasing $h_{0}$ or values of N<2. Interestingly, a value of N=1.5, with the same other parameters as for a hemisphere, gives a good compromise between minimum reflectance and maximum transmittance. This can be attributed to the fact that, with N<2, the structures are more densely packed than for the hemispheres in a hexagonal lattice (see Figure S5 of the SI). Such tunability between reflectance and transmittance could be of particular interest in applications that require the optimization of either characteristics.

As discussed by McCoy et al. [2], another interesting aspect for such structures comes from their ability to increase the average light path length. In fact, increasing the optical path length would increase the light absorption in an absorbing layer of a given thickness. This would be of particular interest for applications involving solar cells; for example, placing such microstructures on top of a solar cell can increase the power conversion efficiency. Such an effect has already been demonstrated with viola and rose petal replicates from PDMS molds [12,13], where the printed resists placed on top of solar cells improved the power conversion efficiency by approximately 6% and 13%, respectively, at normal incidence. In fact, an increase in path length is one of the main mechanisms behind the peacock spider’s super black effect in nature, due to the presence of the absorbing melanin layer under the microlens array [2]. Because this aspect was left out of our current study, further investigation on this matter would be of great interest.

## Data Availability

All the data present in this paper will be made available upon reasonable request. Please contact the corresponding author for further information.

## Supplementary Materials

The following are available online at https://www.mdpi.com/article/10.3390/nano11092298/s1. Section S1: MATLAB^®^ .STL file generation, Figure S1: Example of single solid structures generated from the surface equation Equation (1) using the surf2solid function, Section S2: 3D printer parameters and samples configuration, Table S1: List of parameters used for 3D printing the structures, Figure S2: Strategy for printing multiple structure arrays at the same time, Section S3: Reflectance and transmittance measurements procedure, Figure S3: Example of structures reflectance spectrum derivation, Figure S4: Example of structures transmittance derivation from camera images, Section S4: List of 3D printed geometries and corresponding SEM images, Table S2: List of printed hemispheres ($h_{0}=1$, $e_{0}=1$ and N=2) according to their chacteristic structure size $R_{0}$ and corresponding reflectance (λ=550 nm) and transmittance, Table S3: List of the other printed samples ($R_{0}=5$ µm), corresponding shape parameters from Equation (1) and corresponding reflectance (λ=550 nm) and transmittance, Figure S5: Scanning electron micrograph of representative structures with $R_{0}=5$ µm, Section S5: Estimation of the diffraction efficiency for hemispheres.
