# Supplementary material for: Bioinspired Microstructured Polymer Surfaces with Antireflective Properties
Source: Nanomaterials (Basel). 2021 Sep 4;11(9):2298. doi: 10.3390/nano11092298 (PMC8470586; doi:10.3390/nano11092298)
Supplement: Supplementary file 1 [file nanomaterials-11-02298-s001.zip › nanomaterials-1326150-supplementary.pdf]

Supplementary Materials

Bioinspired Microstructured Polymer Surfaces with Antireflective Properties

Alexandre Emmanuel Wetzel [1], Nuria del Castillo Iniesta [1], Einstom Engay [1], Nikolaj Kofoed Mandsberg [2,3], Celine Schou Dinesen [1], Bilal Rashid Hanif [1], Kirstine Berg-Sørensen [3], Ada-Ioana Bunea [1,*] and Rafael Taboryski [1]

[1] National Centre for Nano Fabrication and Characterization (DTU Nanolab), Technical University of Denmark, Ørsted Plads 347, 2800 Kongens Lyngby, Denmark; awet@dtu.dk (A.E.W.); nurin@dtu.dk (N.d.C.I.), einen@dtu.dk (E.E.); s173957@student.dtu.dk (C.S.D.); s173968@student.dtu.dk (B.R.H.); rata@dtu.dk (R.T.)

[2] Center for Intelligent Drug Delivery and Sensing Using Microcontainers and Nanomechanics (IDUN), Technical University of Denmark, Ørsted Plads 345C, 2800 Kongens Lyngby, Denmark; nikoma@dtu.dk (N.K.M.)

[3] Department of Health Technology (DTU Health Tech), Technical University of Denmark, Ørsted Plads 345C, 2800 Kongens Lyngby, Denmark; kibs@dtu.dk

* Correspondence: adabu@dtu.dk; Tel.: +45-29404623

Abstract:

Over the years, different approaches to obtaining antireflective surfaces have been explored such as using index-matching, interference, or micro- and nanostructures. Structural super black colors are ubiquitous in nature and biomimicry thus constitutes an interesting way to develop antireflective surfaces. Moth-eye nanostructures, for example, are well known and have been successfully replicated using micro- and nanofabrication. However, other animal species such as birds of paradise and peacock spiders have evolved to display larger structures with antireflective features. In peacock spiders, the antireflective properties of their super black patches arise from relatively simple microstructures with lens-like shapes organized in tightly packed hexagonal arrays, which makes them a good candidate for cheap mass replication techniques. In this paper, we present the fabrication and characterization of antireflective microarrays inspired by the peacock spiders' super black structures encountered in nature. Firstly, different microarrays 3D models are generated from a surface equation. Secondly, the arrays are fabricated in a polyacrylate resin by super-resolution 3D printing using two-photon polymerization. Thirdly, the resulting structures are inspected using a scanning electron microscope. Finally, the reflectance and transmittance of the printed structures are characterized at normal incidence with a dedicated optical setup. The bioinspired microlens arrays display excellent antireflective properties, with a measured reflectance as low as 0.042 ± 0.004% for normal incidence, a wavelength of 550 nm, and a collection angle of 14.5°. These values were obtained using a tightly-packed array of slightly pyramidal lenses with a radius of 5 μm and a height of 10 μm.

Keywords: 3D printing, antireflective, biomimetic, polymer microstructures, super black, two-photon polymerization

S1. MATLAB® .STL file generation

To obtain .STL files that can be used by the printer software, single microlens structures were first created according to the surface equation **Eq. 1**. x and y position arrays were generated according to the following parametric equations:

$$x_i = r_i \cos(\phi_j),\ y_i = e_0 r_i \sin(\phi_j) \tag{2}$$

where r_i and ϕ_j were varied between 0 and R_0, and 0 and 2π respectively. The corresponding z positions were then retrieved from **Eq. 1** to combine the 3D positions of a single structure. Furthermore, the single structure was turned into an array by repeating the z positions and adding a constant offset to the x and y positions. Finally, the .STL files were generated using the *surf2solid* and *stlwrite* functions available from Sven Holcombe [1,2]. Examples of the obtained solid single structures, following the use of the *surf2solid* function, can be seen in **Fig. S1**.

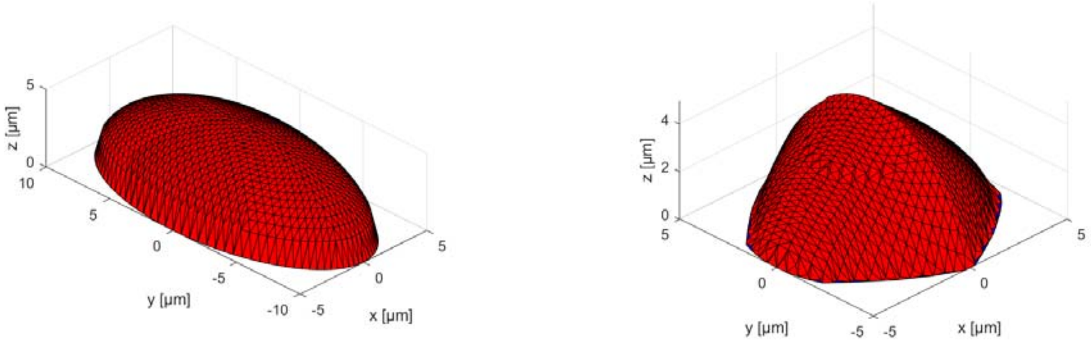

(**a**) (**b**)

Figure S1. Example of single solid structures generated from the surface equation **Eq. 1** using the *surf2solid* function. (a) Structure generated with shape parameters $R_0 = 5$ [μm], $h_0 = 1$, $e_0 = 2$ and $N = 2$; (b) Structure generated with shape parameters $R_0 = 5$ [μm], $h_0 = 1$, $e_0 = 1$ and $N = 1$.

S2. 3D printer parameters and samples configuration

The Nanoscribe 2PP 3D printer allows one to choose between different configurations according to the wanted printed structures features. In our case, it was crucial to print with the highest resolution available of around 200 nm, this, in order to create geometries as close as possible to the theoretical ones. The printed structures were also relatively small in heights. Therefore, the best configuration for the Nanoscribe was when using the IP-L 780 photoresist along with the 63x/1.40 Plan-Apochromat objective (Carl Zeiss, Oberkochen, Germany). With such a configuration, the printer is able to print over around a square of 140 μm of side lengths without having to move the piezo stage. Although it would be beneficial to create a very large array of structures, having to move the piezo stage on the printer is known to induce stitching errors, where some structures on the edges of each repetition can be shifted or deformed. With respect to this, and in order to minimize the printing time of each sample, we limited our printed array sizes to one field of view of the printer's objective. More details on the exact configuration parameters can be observed in **Table S1**.

Table S1. List of parameters used for 3D printing the structures.

Parameter	Setting
Substrate	170 µm thick borosilicate glass coverslips
Slicing mode	Fixed
Slicing distance (µm)	0.2
Contour count	1
Hatching distance	0.2
Hatching angle	0
Hatching angle offset (°)	90
Base count	0
Scan mode	Galvo
Laser power (%)	50
Scan speed (µms^{-1})	10000

Several different microlens arrays were printed at a time, each separated by a large distance making use of the piezoelectric stage available. Because of this approach, labelling of each microarray was necessary. We therefore placed numbers or letters as labels above and below each printed array. In addition, in order to locate the printed samples faster while characterizing their reflectance/transmittance, we printed a frame around each array. The frame lines and the label were printed using a height of 2 µm to minimize the overall printing time. The layout for such printed samples can be seen in **Fig S2**.

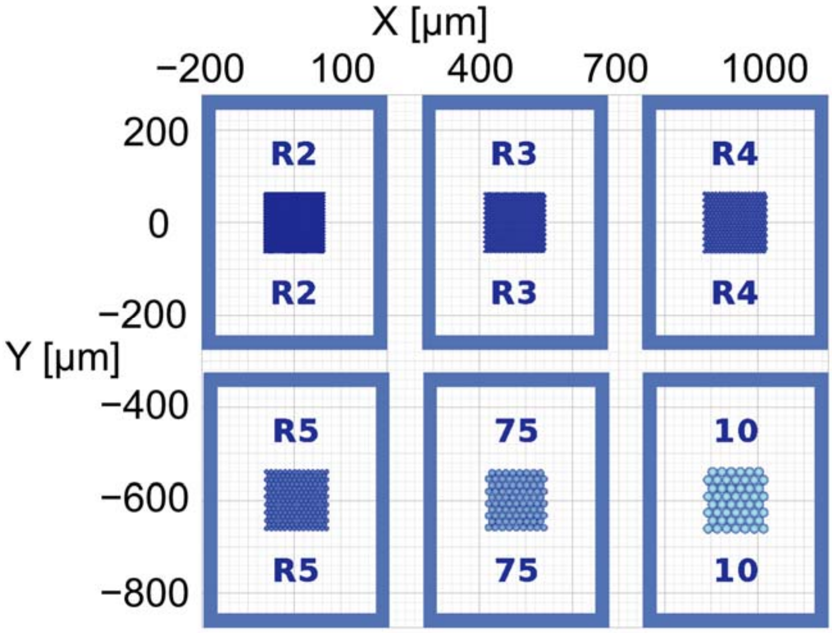

Figure S2. Strategy for printing multiple structure arrays at the same time. Each array is enclosed within a square of 140 µm of side length to respect the maximum field of view (FOV) of the microscope objective. In addition, labels and rectangles are printed around the corresponding arrays to be able to recognize the type of structure printed while measuring their reflectance/transmittance. This example layout was obtained from the Describe software and shows six different arrays of hemispheres with different characteristic structure sizes R_0 from 2 µm (R2) to 10 µm (10).

S3. Reflectance and transmittance measurements procedure

The reflectance measurements were derived using three intensity signals, namely, a reference signal, a dark signal and a signal from the sample itself. The reference signal was obtained by replacing the sample in the setup with a broadband dielectric mirror (Thorlabs BB1-E02), and the dark signal was obtained by measuring the spectrograms

when the light source was turned off. All obtained intensity signals were normalized by the exposure time on the spectrometer sensor, before the reflectance was derived. An example of the normalized intensity signals can be seen in **Fig. S4**.

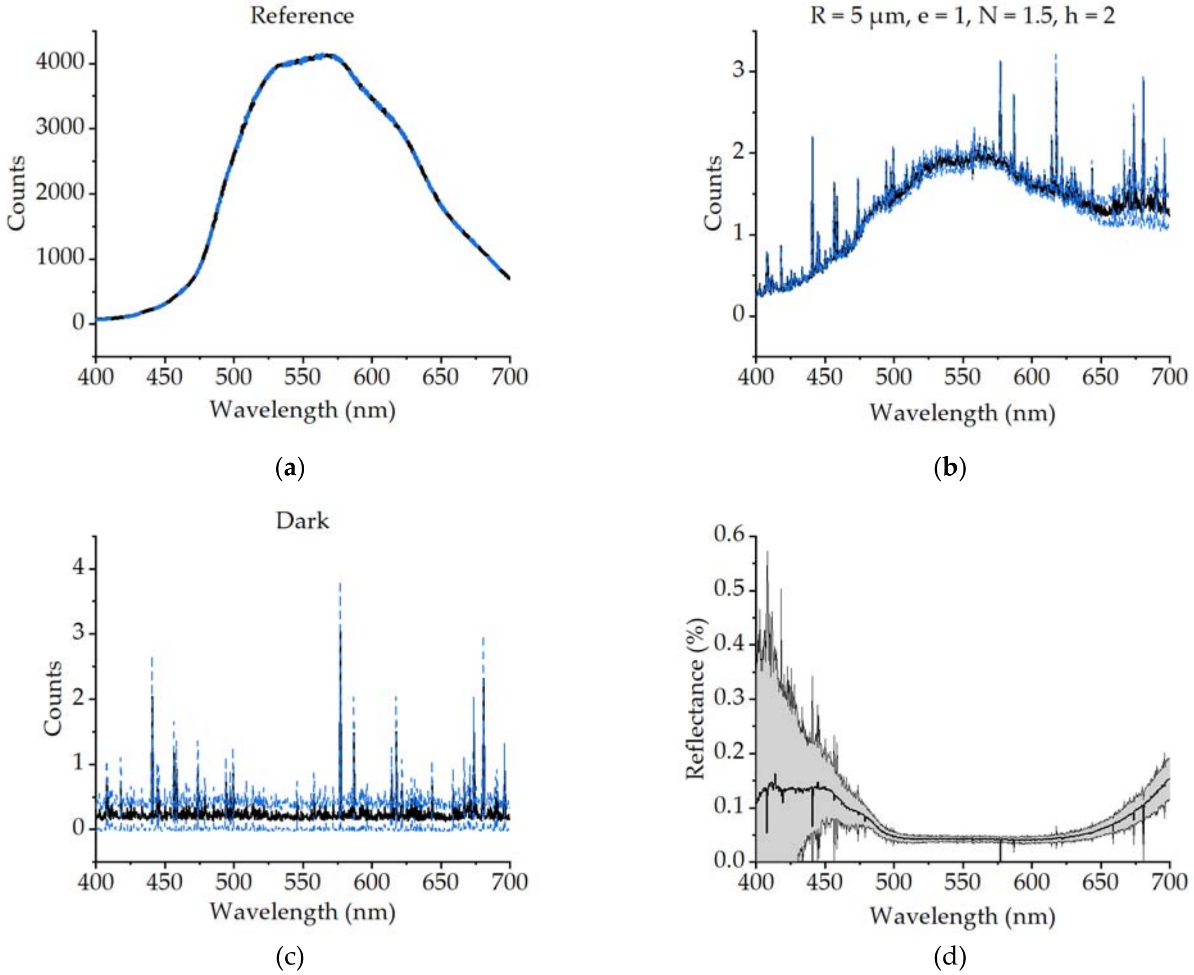

Figure S3. Example of structures reflectance spectrum derivation. (a-c) Reflectance spectrum counts normalized by the exposure time for: (a) a broadband mirror (Thorlabs BB1-E02), (b) a sample with indicated shape parameters, (c) with the light source off (dark); (d) Computed reflectance spectrum following **Eq. 3**.

Similarly, three signals were used to measure the transmittance on the camera. This time, the reference signal was obtained without any samples on the setup, while the dark signal was obtained in the same way as discussed above. All intensity signals were again normalized by the respective exposure time before deriving the transmittance. Since the transmittance was obtained from images, it consists only in an average value over the camera spectral sensitivity. Also, the measurement of the transmittance was limited only to the region in the images where the reference signal was larger than the noise. An example of such images and transmittance as picture representation can be seen in **Fig. S5**.

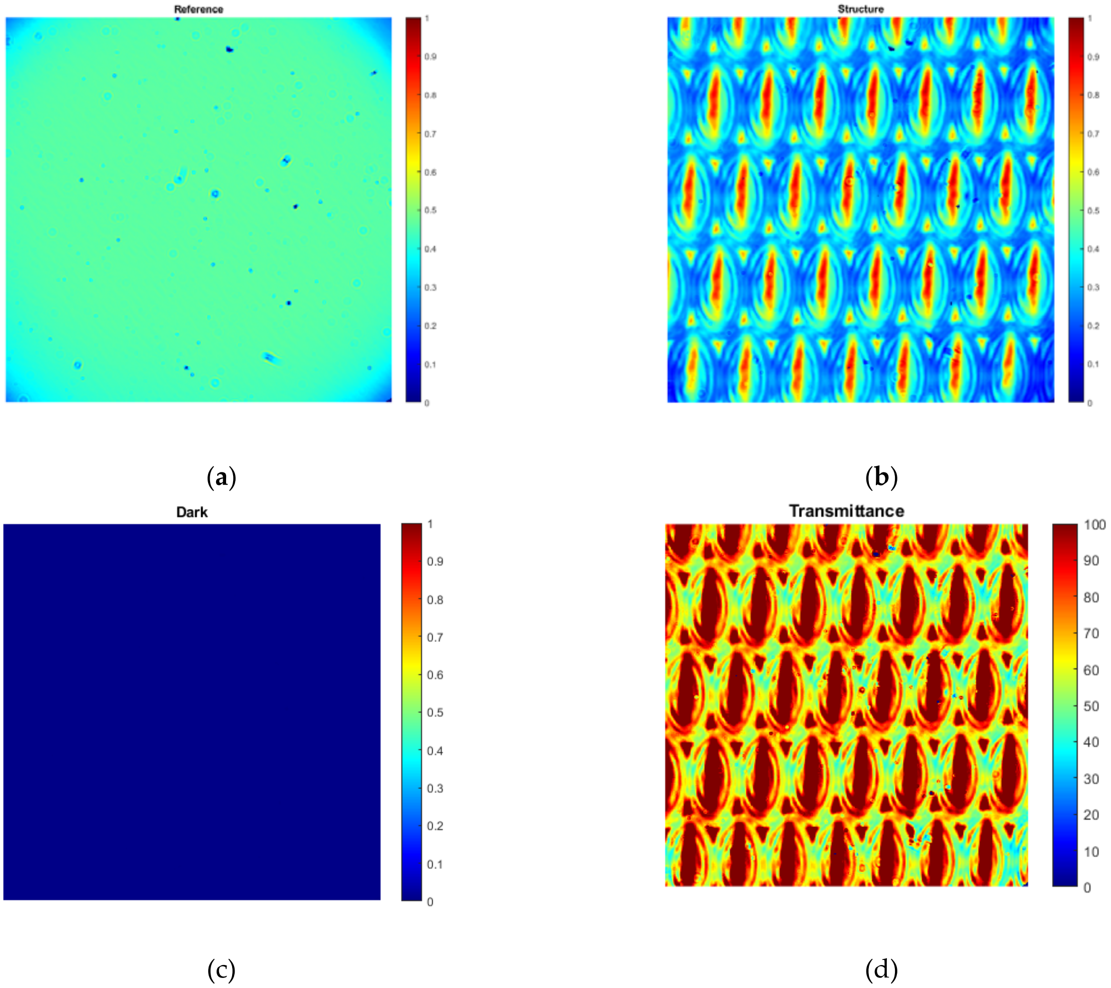

Figure S4. Example of structures transmittance derivation from camera images. (a) Reference image captured with no sample in the setup; (b) Image of the structures; (c) Image with the light source off (dark); (d) Spatial representation of the transmittance in percent. Images (a-c) are here first normalized by the exposure time and then by the maximum value over the three pictures for convenience.

S4. List of 3D printed geometries and corresponding SEM images

All the printed structures (except for a set of flat samples) were printed according to **Eq. 1**. The printed hemispheres sizes, as well as the measured reflectance (at $\lambda = 550$ nm) and transmittance can be found in **Table S2**. Similarly, the shape parameters and characterized reflectance and transmittance for the rest of the samples is shown in **Table S3**. In addition, the SEM pictures from several different samples can be found in **Fig. S5**.

Table S2. List of printed hemispheres ($h_0 = 1$, $e_0 = 1$ and $N = 2$) according to their chacteristic structure size R_0 and corresponding reflectance ($\lambda = 550$ nm) and transmittance.

**R₀ [µm]**	**R [%]**	**T [%]**
2	0.065 ± 0.006	40.9 ± 9.5
3	0.091 ± 0.012	54.3 ± 5.6
4	0.12 ± 0.03	48.4 ± 2.7
5	0.14 ± 0.02	52.7 ± 7.3
7.5	0.16 ± 0.02	51.5 ± 5.0
10	0.234 ± 0.004	55.8 ± 8.5

Table S3. List of the other printed samples ($R_0 = 5$ µm), corresponding shape parameters from **Eq. 1** and corresponding reflectance ($\lambda = 550$ nm) and transmittance.

**h_0 [-]**	**e_0 [-]**	**N [-]**	**R [%]**	**T [%]**
1.5	1	2	0.063 ± 0.006	35.2 ± 5.0
2	1	2	0.046 ± 0.003	26.9 ± 2.3
1	1.5	2	0.195 ± 0.005	74.0 ± 6.1
1	2	2	0.25 ± 0.02	78.6 ± 9.3
1	1	1.5	0.060 ± 0.007	60.8 ± 6.5
1	1	1	0.071 ± 0.009	26.9 ± 4.8
1.5	1	1.5	0.045 ± 0.002	21.4 ± 4.0
2	1	1.5	0.042 ± 0.004	16.3 ± 2.2

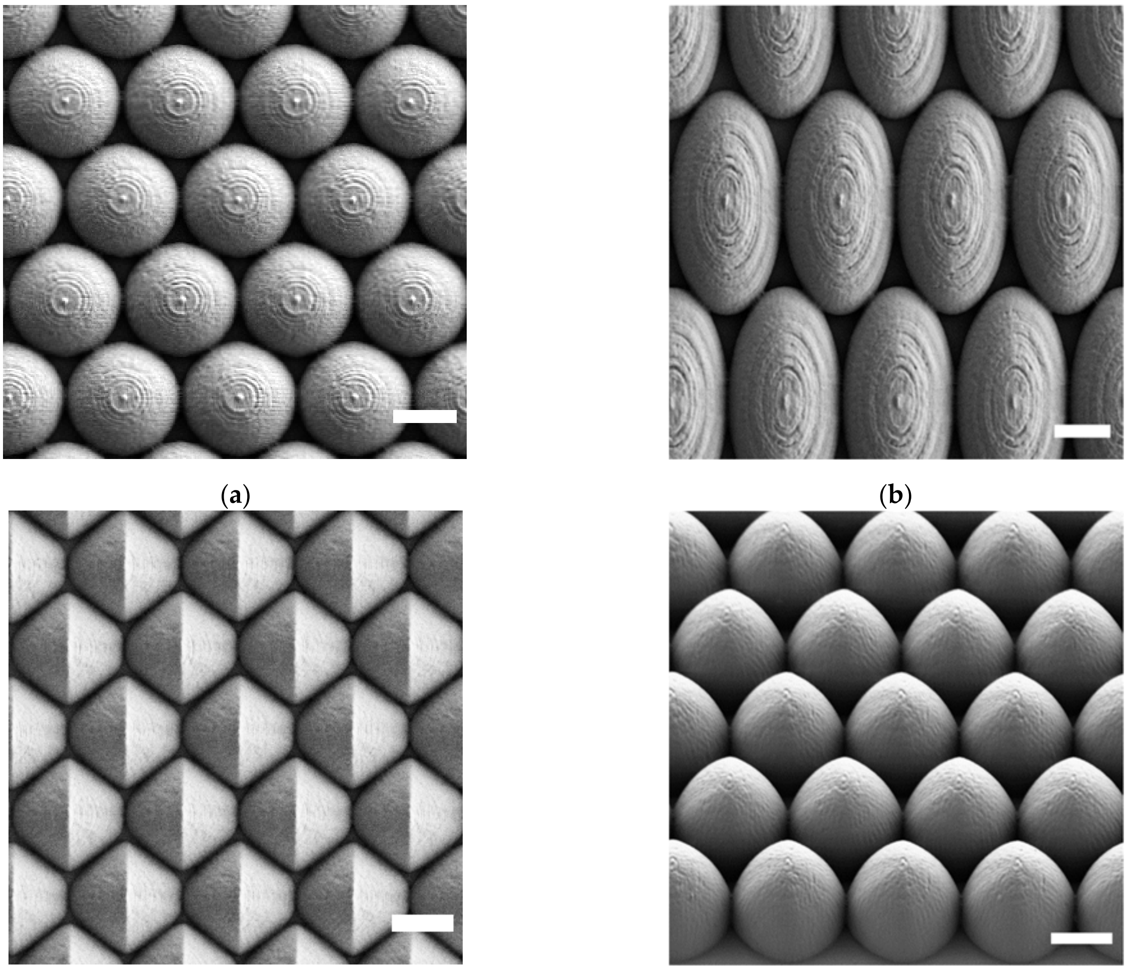

(**a**) (**b**)

(c) (d)

Figure S5. Scanning electron micrograph of representative structures with $R_0 = 5$ µm. (a) Hemispheres $e_0 = 1$, $N = 2$, $h_0 = 1$. (b) Ellipsoidal structures $e_0 = 2$, $N = 2$, $h_0 = 1$. (c) Pyramidal structures $e_0 = 1$, $N = 1$, $h_0 = 1$. (d) Optimized structures $e_0 = 1$, $N = 1.5$, $h_0 = 2$. Images (a-c) were taken at normal incidence while image (d) was tilted by 25° from the normal. Scale bars are 5 µm large.

S5. Estimation of the diffraction efficiency for hemispheres

In order to estimate how much light is collected in our measurements out of the total reflected or transmitted light, we followed the procedure to estimate a gratings diffraction efficiency η based on Fourier coefficients as described by Goodman [3]. Only, we extended the formula for a 2D grating, which gives:

$$c_{u,v} = \frac{1}{\Lambda^2} \iint_{-L/2}^{L/2} P(x,y) e^{-\frac{j2\pi}{\Lambda}(ux+vy)} dxdy \tag{S1}$$

$$\eta_{u,v} = |c_{u,v}|^2 \tag{S2}$$

where $c_{u,v}$ are the 2D Fourier coefficients for diffraction orders u and v, Λ is the grating period, $P(x,y)$ is the grating transmittance function for a unit cell and $\eta_{u,v}$ is the corresponding diffraction efficiency for that particular diffraction order.

We then modelled a hemisphere with radius R_0 as being a thin phase-only element. The grating phase $\varphi(x,y)$ and corresponding transmittance $P(x,y)$ are then:

$$\varphi(x,y) = \frac{2\pi}{\lambda}(n-1)R_0 \left[1 - \left(1 - \frac{x^2+y^2}{{R_0}^2}\right)^{1/2}\right] \tag{S3}$$

$$P(x,y) = e^{j\varphi(x,y)} \tag{S4}$$

where λ is the wavelength and n the refractive index. **Eq. S3** here effectively corresponds to the phase shift for the transmitted light. For the reflected light, the phase function was adapted in the following way:

$$\varphi(x,y)_R = 2\varphi(x,y) - \pi \tag{S5}$$

Furthermore, the diffraction peaks angles were computed from the well-known grating equation:

$$\sin(\theta_u) = \sin(\theta_i) + u\frac{\lambda}{\Lambda} \tag{S6}$$

where θ_i is the incoming light angle, θ_u the diffracted light angle for order u.

For hemispheres, we have that $\Lambda = 2R_0$ and we used $\theta_i = 0$ for a beam coming at normal incidence. In addition, we used $R_0 = 5$ µm and $\lambda = 550$ nm to relate to our measurements, and the refractive index n was estimated for this wavelength based on the work from Gissibl et al. [4]. Integrals for all orders collected by our lens ($2\alpha_R = 14.5°$) or objective lens ($2\alpha_T = 47.2°$), for the reflectance or transmittance measurement respectively were summed in order to get an approximation of the collected light. This resulted in an estimate of 17.8 % and 50.4 % of the total light being collected in our setup, for the reflectance and transmittance respectively. Note that this estimate was made considering a square lattice for the formed structures and did not account for the angular spread of the diffracted light. This means that our estimate here is most likely an overestimate of the light collected in reality.

References

1. Holcombe, S. surf2solid - make a solid volume from a surface for 3D printing 2014.
2. Holcombe, S. stlwrite - write ASCII or Binary STL files 2011.
3. Goodman, J.W. Introduction to Fourier optics. *Introd. to Fourier Opt. 3rd ed., by JW Goodman. Englewood, CO Roberts \& Co. Publ. 2005* **2005**, *1*.
4. Gissibl, T.; Wagner, S.; Sykora, J.; Schmid, M.; Giessen, H. Refractive index measurements of photo-resists for three-dimensional direct laser writing. *Opt. Mater. Express* **2017**, *7*, 2293, doi:10.1364/ome.7.002293.
